# Novel insights into the hepatoprotective mechanisms of SGLT2 inhibitor empagliflozin in Zucker diabetic fatty rats

**DOI:** 10.3389/fphar.2025.1649630

**Published:** 2025-09-23

**Authors:** F. Comella, A. Aragón-Herrera, S. Melini, N. Opallo, S. Feijóo-Bandín, N. P. Navatti, G. Mattace Raso, O. Gualillo, C. Pirozzi, F. Lago-Paz, R. Meli

**Affiliations:** ^1^ Department of Pharmacy, School of Medicine, University of Naples Federico II, Naples, Italy; ^2^ Cellular and Molecular Cardiology Research Unit, Institute of Biomedical Research of Santiago de Compostela (IDIS), Hospital Clínico Universitario de Santiago de Compostela, Área Sanitaria de Santiago de Compostela y Barbanza (SERGAS), Santiago de Compostela, Spain; ^3^ Centro de Investigación Biomédica en Red de Enfermedades Cardiovasculares (CIBERCV), Instituto de Salud Carlos III, Madrid, Spain; ^4^ SERGAS (Servizo Galego de Saude) and IDIS (Instituto de Investigación Sanitaria de Santiago), The NEIRID Group (Neuroendocrine Interactions in Rheumatology and Inflammatory Diseases), Santiago University Clinical Hospital, Building C, Travesía da Choupana SIN, Santiago de Compostela, Spain; ^5^ International PhD School, University of Santiago de Compostela (EDIUS), Santiago deCompostela, Spain

**Keywords:** metabolic-associated fatty liver disease (MAFLD), ZDF rat, glucose and lipidmetabolism, insulin sensitivity, hepatic inflammation, macrophage polarization, fibrosis, pro-resolving mediators

## Abstract

The sodium-glucose cotransporter (SGLT)2 inhibitor empagliflozin (EMPA) is a hypoglycemic drug for patients with type 2 diabetes mellitus and cardiovascular disease. The mechanisms underlying the beneficial effects of EMPA in counteracting Metabolic Associated Fatty Liver Disease (MAFLD) are poorly understood. Our study aimed to evaluate the therapeutic mechanisms of EMPA treatment (30 mg/kg/day in drinking water for 6 weeks) on hepatic dysfunction observed in diabetic obese Zucker Diabetic Fatty (ZDF) rats. EMPA activated hepatic insulin signaling, increasing the phosphorylation of insulin receptor, AKT and AMP-activated protein kinase, and downregulated the expression of gluconeogenesis-related genes (glucose-6-phosphatase and phosphoenolpyruvate carboxykinase). In the liver of EMPA-treated rats, no difference in SGLT2 and SGLT1 expression was found, while a significant upregulation of GLUT2 protein levels suggested other converging mechanisms on hepatoprotective effects of EMPA. Moreover, EMPA improved hepatic lipid metabolism in ZDF rats, modulating key mediators of fatty acid metabolism and catabolism (cluster of differentiation 36, forkhead box protein O1, fatty acid binding protein 1) and mitochondrial function (uncoupling protein 2 and the mitochondrial transporter ATP-binding cassette 1). Then, we demonstrated EMPA effect against hepatic inflammation and fibrosis, associated with insulin resistance, and, for the first time, its potential as pro-resolving agent increasing immune cell recruitment along with the induction of resolvins (annexin A1 and IL-10). Taken together, our study provides new perspectives for EMPA as a multifaceted approach to counteract MAFLD in obesity and diabetes.

## 1 Introduction

Obesity and type 2 diabetes mellitus (T2DM) are major public health challenges and key drivers of metabolic dysfunction-associated fatty liver disease (MAFLD) (Eslam et al., 2020). MAFLD was recently introduced as a broader and clinically relevant concept to replace non-alcoholic fatty liver disease (NAFLD). Unlike NAFLD, MAFLD does not require the exclusion of alcohol consumption or other liver diseases. Instead, it is inclusion-based, meaning it is diagnosed when hepatic steatosis is present along with at least one metabolic risk factor (e.g., obesity, type 2 diabetes, or metabolic syndrome), better reflecting real-world patient profiles. Insulin resistance (IR), lipotoxicity, and chronic low-grade inflammation play critical roles in the progression of MAFLD, increasing the risk of hepatic steatosis, fibrosis, and even hepatocellular carcinoma (Mantovani and Dalbeni, 2021). Given the rising prevalence of obesity-related liver disorders, identifying effective pharmacological interventions with metabolic and hepatoprotective properties is of paramount importance.

Empagliflozin (EMPA), a sodium-glucose cotransporter (SGLT)2 inhibitor, is widely used as an anti-hyperglycemic agent. Large-scale clinical trials have demonstrated a lower incidence of the primary merged cardiovascular outcome and of death from any cause when added to standard therapy in patients with T2DM at high cardiovascular risk (Zinman et al., 2015). Beyond its canonical role in glycemic control, experimental studies support the beneficial effects of EMPA in liver of mice feeding a high fat diet, improving lipogenesis, beta-oxidation, and endoplasmic reticulum stress pathways (Petito-da-Silva et al., 2019). Preclinical and clinical studies confirmed that SGLT2 inhibitors reduce hepatic steatosis, fibrosis, and inflammation through lipid metabolism, enhance mitochondrial function, and reduce endoplasmic reticulum stress (Taheri et al., 2020; Sattar et al., 2021).

A pivotal step in the progression from simple steatosis to MAFLD with clinically relevant outcomes is the persistence of hepatic inflammation, namely, steatohepatitis, when steatosis is associated with inflammatory cell infiltration and progressive fibrosis (Loomba and Sanyal, 2013). This process is strongly influenced by macrophage polarization. Evidence indicates that chronic low-grade inflammation is a cause of hepatic and systemic IR, in which tissue macrophages are central players (Lee and Olefsky, 2021; Puschel et al., 2022). In the inflamed liver, macrophages predominantly exhibit an M1 pro-inflammatory phenotype, releasing cytokines such as tumor necrosis factor (TNF)-α and interleukin (IL)-6, which contribute to hepatocyte injury and fibrosis (Kazankov et al., 2019). Conversely, M2 macrophages exhibit an anti-inflammatory and pro-resolving phenotype, secreting cytokines such as interleukin (IL)-10 and transforming growth factor (TGF)-β, promoting tissue repair and fibrosis resolution (Wang et al., 2021). Promoting the shift of macrophages from the pro-inflammatory M1 to the anti-inflammatory M2 phenotype could be crucial for resolving hepatic inflammation and preventing disease progression (Cheng et al., 2021). Specialized pro-resolving mediators (SPMs) represent an emerging class of endogenous molecules that actively drive the resolution of inflammation. Resolvins, in particular, enhance macrophage polarization toward the M2 phenotype, reduce chronic inflammatory signaling, and promote tissue repair and fibrosis resolution (Titos et al., 2011; Musso et al., 2018; Serhan et al., 2018). Given the emerging evidence suggesting that SGLT2 inhibitors can modulate inflammation and oxidative stress (Winiarska et al., 2021; Schonberger and Tchorz, 2023; Zheng et al., 2025), their potential role in macrophage polarization and resolvin-mediated hepatic protection requires further investigation.

The Zucker Diabetic Fatty (ZDF) rat, developing obesity, hyperinsulinemia, hyperglycemia, IR, and hyperlipidemia, is a valuable animal model for studying key aspects of human metabolic syndrome and evaluating the efficacy and mechanisms of potential therapeutic agents (Forcheron et al., 2009; Wang et al., 2014; Zhou et al., 2020).

Here, we investigated the hepatoprotective effects of EMPA in ZDF rats, focusing on its ability to improve hepatic glucose and lipid metabolism and to promote inflammation resolution through endogenous pro-resolving mediators. Our findings provide novel insights into the therapeutic potential of EMPA for the treatment of MAFLD.

## 2 Materials and methods

### 2.1 Animal care

ZDF rats (ZDF-Lepr^*fa/fa*^) are used as a model for early-stage T2DM, characterized by high insulin levels and glucose intolerance in the liver and skeletal muscle (Shiota and Printz, 2012). Male ZDF rats weighing between 200 and 250 g were sourced from Charles River Laboratories (United States). To induce programmed and consistent development of T2DM, the rats were fed *ad libitum* with the diabetogenic Formulab 5008 diet (LabDiet, United States) following the supplier’s instructions, starting at 7 weeks of age. Characteristics of the male ZDF rat maintained on Formulab 5008 diet include hyperinsulinemia and hyperglycemia, T2DM, insulin resistance and obesity. The animals were housed in the Animal House of the CiMUS (Centro Singular de Investigación en Medicina Molecular y Enfermedades Crónicas, Santiago de Compostela, Spain) under controlled conditions, including a room temperature of 22 °C ± 2 °C, relative humidity of 40%–50%, and a 12-h light/12-h dark cycle, with unrestricted access to chow and water. This work is a complementary examination of the hepatic effects of empagliflozin from our previous studies in heart, liver and adipose tissue (Aragon-Herrera et al., 2019; Aragon-Herrera et al., 2022; Aragon-Herrera et al., 2023).

### 2.2 Empagliflozin in vivo treatment

When the ZDF rats, fed a diabetogenic diet, reached fasting glucose levels of 350.75 ± 18.59 mg/dL at 12 weeks of age, they were randomly divided into two groups: a control group receiving vehicle (mineral drinking water, ZDF, n = 6) and a treated group receiving 30 mg/kg/day of EMPA (Boehringer Ingelheim Pharma GmbH and Co. KG, DEU) in the drinking water (ZDF + EMPA, n = 6) (Aragon-Herrera et al., 2019). After 6 weeks of treatment, blood samples were collected from all experimental groups. Then, the animals were euthanized, and the livers were collected between 9:00 and 11:00 a.m., quickly frozen in liquid nitrogen, and stored at −80 °C. Livers employed in this study were the same employed in our previous research at the hepatic level (Aragon-Herrera et al., 2022).

### 2.3 Biochemical determinations

Fasting glucose was measured employing a glucometer GlucoDr auto™ (All Medicus Co. Ltd. KOR). Plasma was obtained by centrifugation at 2000 rpm for 10 min at room temperature and subsequently stored at −80 °C until analysis. Plasma insulin concentration was determined using the Ultra Sensitive Rat Insulin ELISA Kit (#90060) (Crystal Chem, NL) following the manufacturer’s instructions. The Homeostasis Model Assessment of Insulin Resistance (HOMA-IR) was calculated using the formula: HOMA-IR = [fasting glucose (mmol/L) × fasting insulin (μU/mL)]/22.

### 2.4 Western blot analysis

Total protein lysates from liver tissues were processed by SDS-PAGE, as previously described (Pirozzi et al., 2023). Proteins were transferred onto membranes using the Trans-Blot Turbo Transfer System (Bio-Rad Laboratories, Segrate, Milan, Italy) for 60 min at 240 mA at room temperature. Membranes were blocked for 60 min at room temperature in 1X phosphate-buffered saline (PBS) containing 5% non-fat dry milk. Subsequently, membranes were incubated with the following primary antibodies: anti-phospho-insulin receptor (InsR)β mouse polyclonal antibody (#44809G, dilution 1:1000) (Thermo Fisher Scientific Inc., Segrate, Milan, Italy), anti-InsRβ rabbit monoclonal antibody (#3025, dilution 1:1000), anti-phospho-AKT (Ser473) rabbit monoclonal antibody (#4060, dilution 1:1000), anti-AKT (pan) mouse monoclonal antibody (#4691, dilution 1:1000), anti-phospho-AMPKα rabbit monoclonal antibody (#2535, dilution 1:1000), and anti-AMPKα mouse monoclonal antibody (#2793, dilution 1:1000) (Cell Signaling Technology, Inc., Beverly, MA, United States), anti-GLUT2 mouse monoclonal antibody (#sc518022, dilution 1:500), anti-SGLT2 (#sc-393350, dilution 1:500) (Santa Cruz Biotechnology, Heidelberg, Germany), and anti-SLC5A1 (SGLT1, #DFT2022, dilution 1:500) (Affinity Biosciences). GAPDH rabbit polyclonal antibody (MAB374, dilution 1:8000) (Merk Life Science S. r.l., Milan, Italy) was used as housekeeping. Signal detection was performed using an enhanced chemiluminescence (ECL) system (Pierce, Thermo Fisher Scientific, #32109, Rodano (MI), Italy) and visualized with the ChemiDoc Imaging System (#12003153, Bio-Rad Laboratories, Segrate, Milan, Italy).

### 2.5 Semi-quantitative real-time PCR analysis

Total RNA from livers was extracted using the PureZOL™ RNA Isolation Reagent (#7326890, Bio-Rad Laboratories, Segrate, Milan, Italy) according to the protocol provided by the RNA extraction kit (#740955.250, NucleoSpin^®^, Macherey-Nagel GmbH and Co., Düren, Germany). cDNA was synthesized from 8 μg of total RNA using the High-Capacity cDNA Reverse Transcription Kit (#4374966, Applied Biosystems, Foster City, CA, United States).

RT-PCR was performed using a Bio-Rad CFX96 Connect Real-Time PCR System and its associated software (Bio-Rad Laboratories, Milan, Italy), under conditions previously described (Lama et al., 2021). Each reaction contained 500 ng of cDNA in 2X QuantiTect SYBR Green PCR Master Mix (# 204145, Qiagen, Hilden, Germany) and specific primer pairs for amplifying the following genes: Interleukin (Il)-1β (*Il1b*, QT00181657), tumor necrosis factor (TNF)-α (*Tnf*, QT00178717), Il-10 (*Il10*, QT00177618), peroxisome proliferator-activated receptor (PPAR)-γ (*Pparg*, QT00186172), PPAR-γ coactivator (PGC)-1α (*Ppargc1a*, QT00189196), Forkhead box protein O1 (FOXO-1) (*Foxo1,* QT00446943), sterol regulatory element binding transcription factor (SREBP)-1 (*Srebf1*, QT00432684), Fatty acid binding protein (FABP)-1 (*Fabp1*, QT00188783), ATP binding cassette subfamily G member (ABCG)-1 (*Abcg1*, QT00176533), glucose-6-phosphatase (G6P) (*G6pc*, QT00185948), phospho-enol pyruvate carboxykinase (PCK) (*Pck1*, QT01619975), transforming growth factor (TGF)β (*Tgfb1,* QT00187796), type I collagen alpha 1 chain (*Col1a1*, QT01081059), type III collagen alpha 1 chain (*Col3a1*, QT01083537), uncoupling protein (UCP)-2 (*Ucp2*, QT00186508), Il-34 (*Il34*, QT01574601), T Cell Immunoglobulin And Mucin Domain Containing (TIMD)4 (*Timd4*, QT02546803), Cluster of differentiation (CD) 163 (*Cd163*, QT02542967), annexin A (*Anxa1*, QT00179361), Integrin subunit alpha X (ITGAX) (*Itgax*, QT00542668), chemokine (C-C motif) ligand 2 also referred as monocyte chemoattractant protein (MCP)1 (*Ccl2*, QT00183253), Solute Carrier Family 5 Member 1 (SGLT1) (*Slc5a1*, QT00177247), Solute Carrier Family 5 Member 2 (SGLT2) (*Slc5a2*, QT00180852) (Qiagen, Hilden, Germany), in a final volume of 50 μL. The relative mRNA levels were normalized to Actin (*Actb,* QT00193473) (Qiagen, Hilden, Germany) as the housekeeping gene, and the data were analyzed using the 2^−ΔΔCT^ method.

### 2.6 Statistical analysis

All data are presented as mean value ± Standard Error of the Mean (S.E.M.). Statistical analysis was performed by Student’s t-test. Differences between groups were considered significant at values of *p* < 0.05. Analyses were performed using GraphPad Prism 10 (GraphPad Software, San Diego, CA, United States).

## 3 Results

### 3.1 EMPA improves insulin resistance and hepatic insulin sensitivity in ZDF rats

**FIGURE 1:**
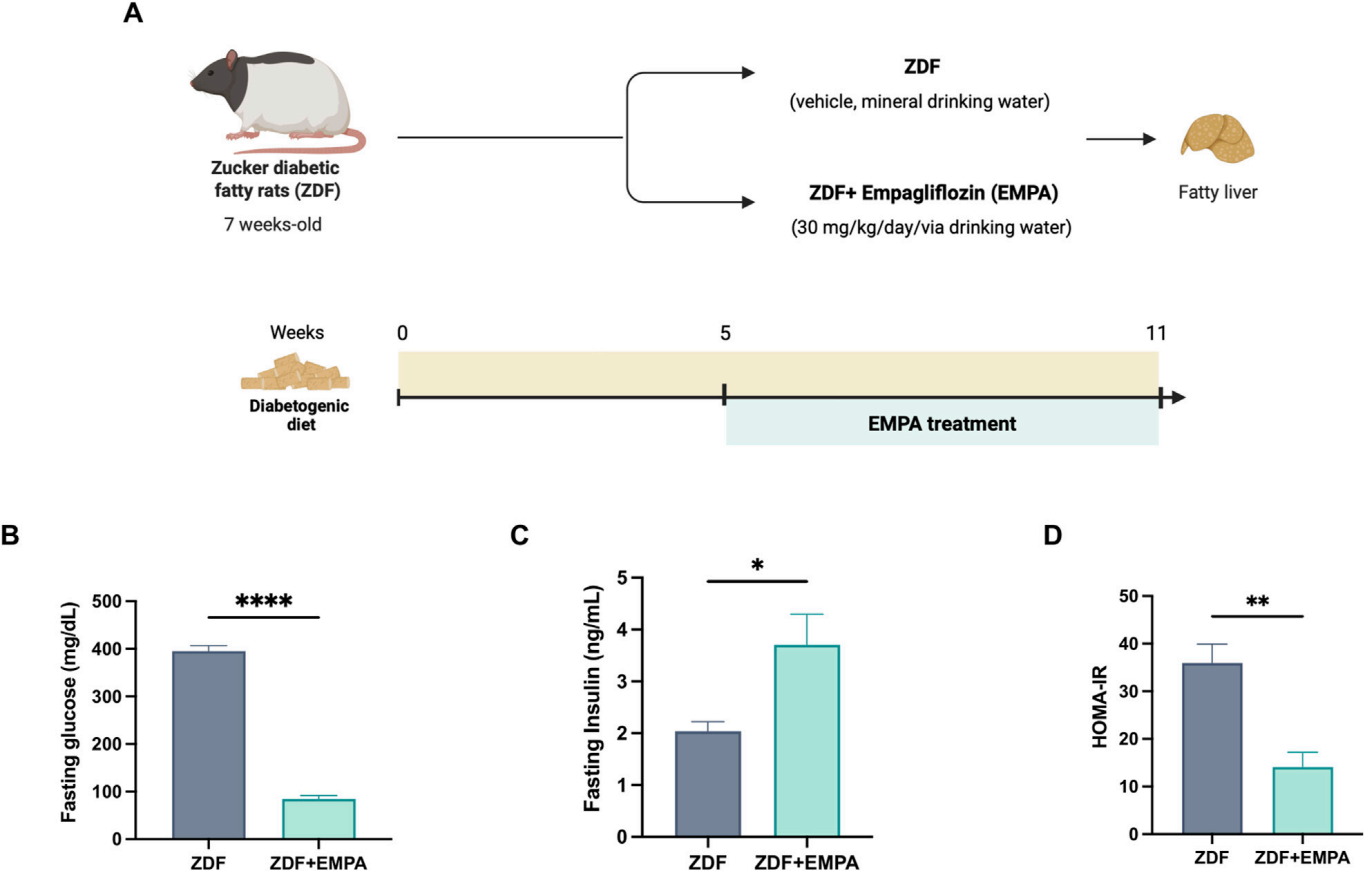
Effects of EMPA on hyperglycemia and insulin resistance in ZDF rats. Schematic representation of the experimental design created in BioRender https://BioRender.com/qn3bexw **(A)**, Fasting glucose **(B)** and insulin **(C)** levels were measured in the plasma of all mice. Insulin resistance was evaluated *ex vivo* by HOMA-IR index **(D)** (n = 4-5 per group). Data are presented as mean ± S.E.M. *p < 0.05, **p < 0.01, and ****p < 0.0001 vs. ZDF group.

The beneficial effect of EMPA in limiting IR in diabetic obese rats, in our experimental conditions (Figure 1A), is primarily shown by the reduction of fasting glucose (Figure 1B) and the increase of insulinemia (Figure 1C), and confirmed by reduction of HOMA-IR index (Figure 1D).

**FIGURE 2:**
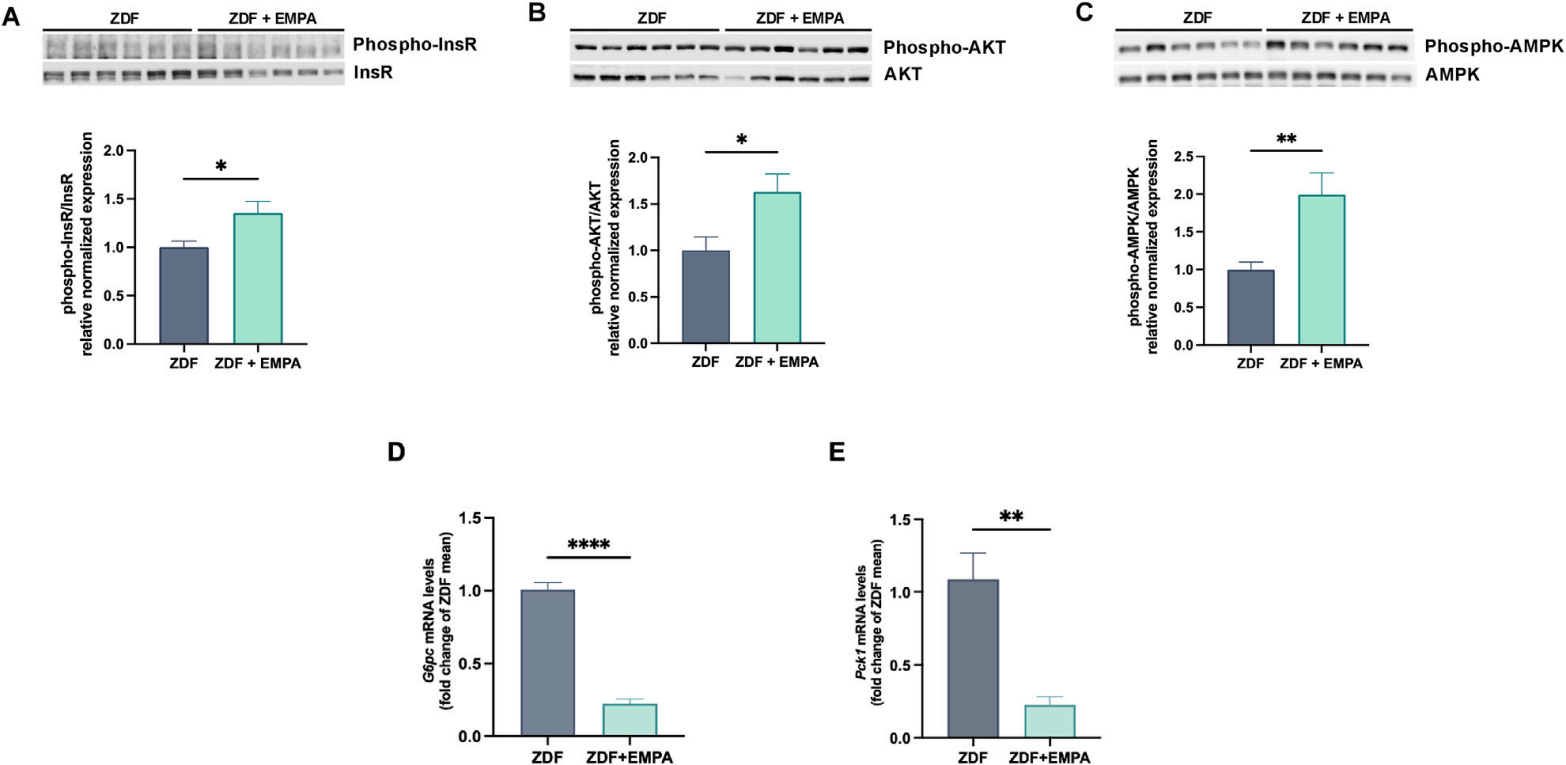
Impact of EMPA on hepatic insulin signaling in diabetic obese rats. Western blot images showing the phosphorylation levels of InsR **(A)**, AKT **(B)**, and AMPK **(C)** in the liver. Hepatic mRNA expression levels of *G6pc* **(D)** and *Pck1* **(E)** were quantified by RT-PCR. Data (n = 6 per group) are presented as mean ± S.E.M. *p < 0.05, **p < 0.01, and ****p < 0.0001 vs. ZDF group.

In the liver, EMPA treatment improved insulin sensitivity by activating key pathways of the insulin signaling. Indeed, EMPA increased the phosphorylation of InsR (Figure 2A) and activated the downstream AKT pathway (Figure 2B). Concomitantly, EMPA also increased AMPK phosphorylation in the liver of ZDF rats (Figure 2C). This kinase represents a key regulator of cellular and tissue energy homeostasis, involved in both lipid and glucose metabolism, restoring liver insulin sensitivity. Finally, EMPA significantly reduced G6P and PEPCK gene expression, two crucial hepatic enzymes involved in gluconeogenesis (Figures 2D,E).

### 3.2 Effect of EMPA on hepatic SGLTs and GLUT2 expression in ZDF animals

**FIGURE 3:**
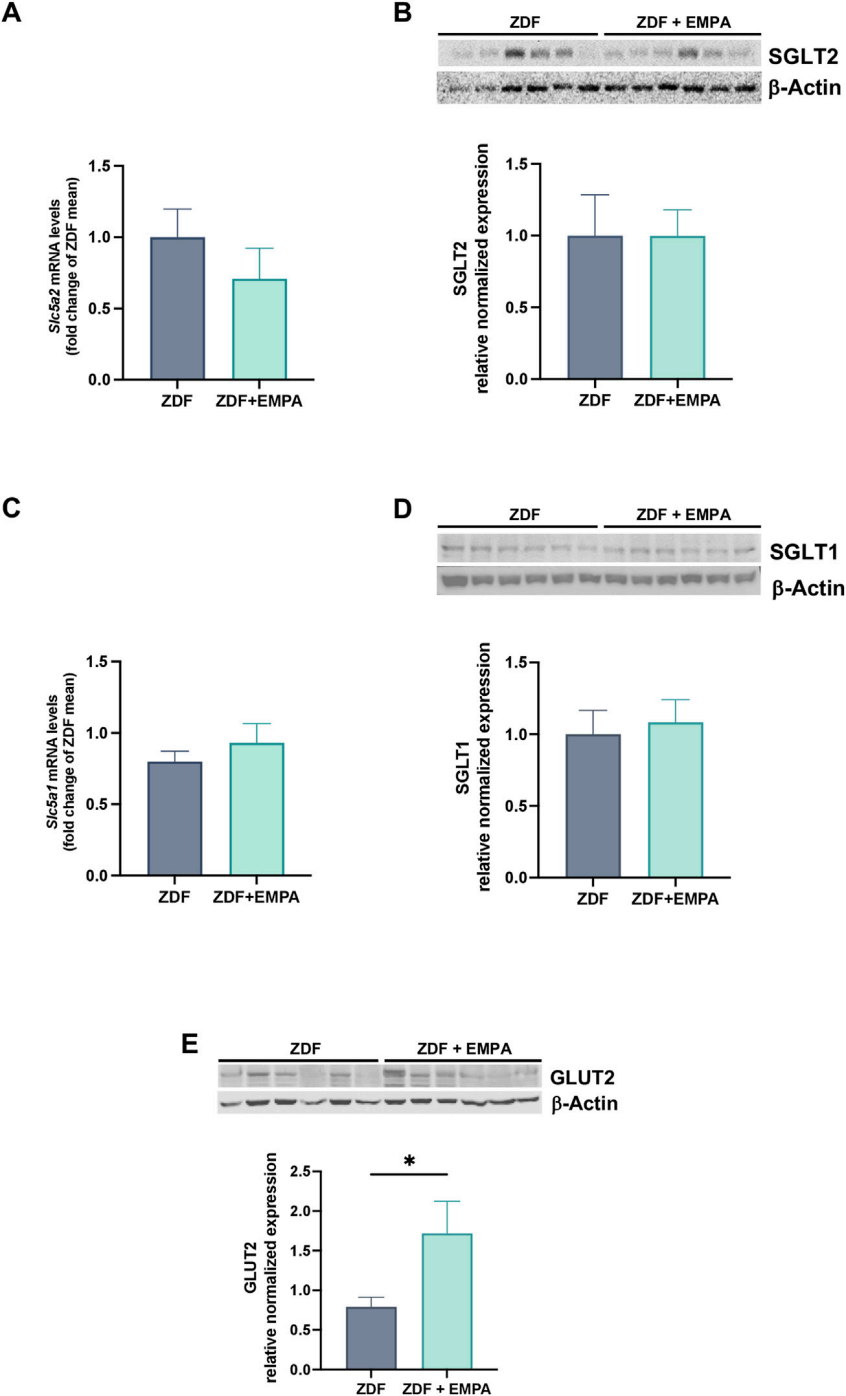
EMPA modulation of glucose transporters in the liver of treated animals. Hepatic gene expression of *Slc5a2* (SGLT2) **(A)** and *Slc5a1* (SGLT1) **(C)**. Immunoblots of hepatic protein levels of SGLT2 **(B)** and SGLT1 **(D)**, and GLUT2 **(E)** in mice. Data (n = 6 per group) are presented as mean ± S.E.M. *p < 0.05 vs. ZDF group.

The mRNA and protein expression of co-transporter SGLT1 and SGLT2 in the liver of ZDF rats treated or not with EMPA were examined. No difference was found in the transcriptional and protein expression of both SGLT2 (Figures 3A,B) and SGLT1 (Figures 3C,D) between EMPA-treated and untreated rats. Notably, although EMPA did not modulate the expression of SGLT co-transporters, drug treatment increased hepatic GLUT2 protein expression (Figure 5E) suggesting a regulatory role of EMPA on insulin-mediated glucose transport in the liver, that is consistent with its hypoglycemic effect.

### 3.3 EMPA ameliorated lipid metabolism and mitochondrial function altered in ZDF rats

**FIGURE 4:**
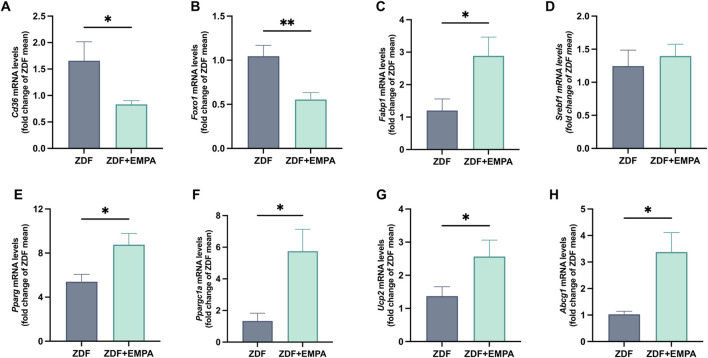
Effects of EMPA treatment on hepatic lipid metabolism of ZDF rats. Evaluation of hepatic mRNA expression levels of *Cd36* **(A)**, *Foxo1* **(B)**, *Fabp1* **(C)**, and *Srebf1* **(D)**, quantified by RT-PCR. mRNA levels of mitochondrial biogenesis and function markers *Pparg* **(E)**, *Ppargc1a* **(F)**, *Ucp2* **(G)**, and *Abcg1* **(H)** were also assessed. Data (n = 6 per group) are presented as mean ± S.E.M. *p < 0.05 and **p < 0.01 vs. ZDF group.

EMPA treatment counteracted lipid dysmetabolism of ZDF rats. It reduced the gene expression of CD36 and FoxO1, two key markers of steatosis involved in *de novo* lipogenesis (Figures 4A,B), and increased FABP1, a regulator of fatty acid trafficking contributing to prevent hepatic lipotoxicity (Figure 4C). No change in the transcription of SREBP-1c between experimental groups was found (Figure 4D). Furthermore, EMPA significantly increased the expression of hepatic PPARγ and its coactivator PGC1α (Figures 4E,F), as well as UCP2 and ABCG1 (Figures 4G,H), all regulatory components of mitochondrial energetic adaptations.

### 3.4 Hepatic anti-inflammatory and pro-resolving effects of EMPA in ZDF rats

**FIGURE 5:**
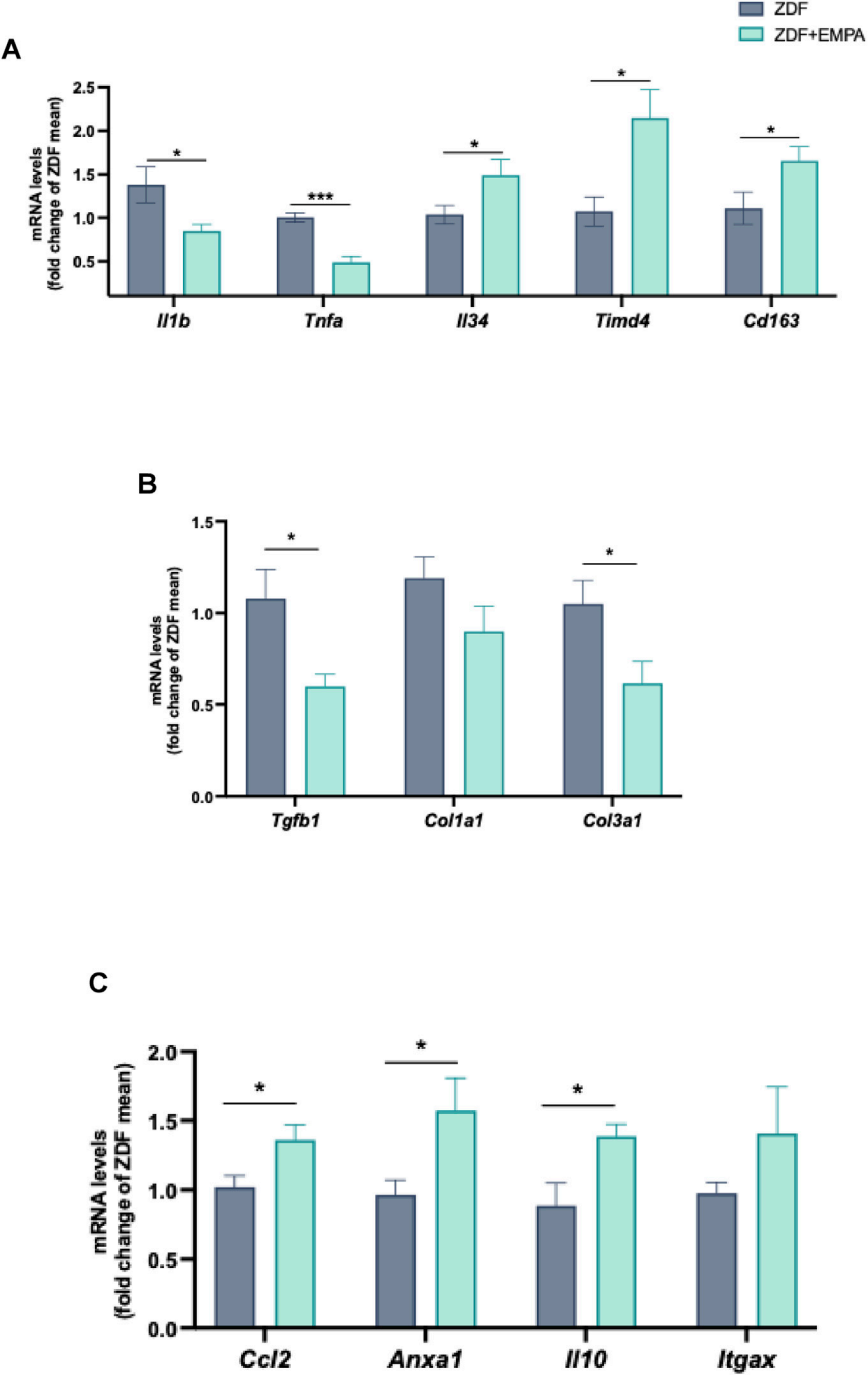
Anti-inflammatory and pro-resolving actions of EMPA in the liver of ZDF rats. RT-PCR analysis of Il1b, Tnfa, Il34, Timd4, Cd163 **(A)** mRNA expression in liver tissue. mRNA levels of fibrotic genes Tgfb, Col1a1, and Col3a1 **(B)**, as well as pro-resolving factors Ccl2, Anxa1, Il10, and Itgax **(C)** were also evaluated. Data (n = 6 per group) are presented as mean ± S.E.M. *p < 0.05 and ***p < 0.001 vs. ZDF group.

EMPA showed beneficial effect against the hepatic inflammation mainly associated with IR and concurrent hyperglycemia characterizing this animal model. Specifically, EMPA markedly reduced the gene expression of pro-inflammatory mediators, such as TNF-α and IL-1β (Figure 5A), and increased the expression of IL-34, TIMD4, and CD163 (Figure 5A), all markers of M2 macrophage polarization. Moreover, EMPA treatment limited the hepatic fibrotic progression in diabetic obese rats, reducing significantly the mRNAs of pro-fibrotic factors such as TGFβ and type III collagen, without modifying type I collagen (Figure 5B). Notably, EMPA exerted a pro-resolving effect as demonstrated by the significant increase in hepatic monocyte recruitment (MCP-1 mRNA) (Figure 5C) and the so-called resolvin AnxA1, as well as the anti-inflammatory cytokine IL-10 (Figure 5C). AnxA1 is an important factor involved in the resolution of inflammation in different disorders including diabetes, obesity and steatohepatitis. Finally, a trend of change was observed for the chemokine Cd11c (or *Itgax*) (Figure 5C).

## 4 Discussion

The present study provides novel insights into the hepatoprotective effects of EMPA in the Zucker obese rat as well-established and translationally relevant preclinical model for investigating MAFLD-related hepatic diseases (Wang et al., 2014; Zhou et al., 2020; Tovar et al., 2024). ZDF rats exhibit key metabolic features of human T2DM, such as hyperinsulinemia, glucose intolerance, and lipid dysregulation, making them an ideal model for studying the impact of pharmacological interventions on liver dysfunction (Shiota and Printz, 2012). Furthermore, the chronic inflammatory state observed in this model closely resembles the pathophysiology of human MAFLD, reinforcing its utility in assessing the anti-inflammatory and pro-resolving effects of EMPA.

A key finding of our study is that EMPA treatment enhances insulin sensitivity of ZDF rats, as shown by the reduction of HOMA-IR index and the increased hepatic phosphorylation of the insulin receptor and AKT along with AMPK activation. The increase in the hepatic pAKT/AKT ratio observed after EMPA treatment reflects the restoration of insulin signal transduction in ZDF rats. This effect is likely mediated by enhanced insulin receptor phosphorylation, reduced gluconeogenic drive, alleviation of lipotoxic and inflammatory stress, and the upregulation of GLUT2, which together favor AKT activation. These findings are consistent with previous reports showing that SGLT2 inhibitors re-establish hepatic AKT signaling and improve insulin sensitivity in metabolic disease models and patients (Xu et al., 2017; Taheri et al., 2020; Sattar et al., 2021; Yu et al., 2022).

Preclinical studies reported that EMPA treatment protects against hepatic steatosis and IR by increasing energy expenditure, adipose tissue browning and improving muscle mitochondrial morphology or favorably modulating intestinal bacteria composition in nutritional rodent models independently of diet type (Xu et al., 2019; Radlinger et al., 2023; Huang et al., 2024). In the liver, insulin stimulates aerobic and anaerobic glucose metabolism and its storage as glycogen, inhibits glucose production directly by inactivating glycogen phosphorylase and by suppressing gluconeogenic enzymes (Puschel et al., 2022). Here, EMPA reduced G6P and PEPCK transcription, two crucial hepatic enzymes involved in gluconeogenesis, undoubtedly linked to the improvement of insulin sensitivity in liver (Yu et al., 2021; Tan et al., 2025). These findings align with previous data in *db/db* mice, where EMPA maintained glucose homeostasis by suppressing gluconeogenesis and enhancing glycogenesis via activation of AMPK/CREB/GSK3β signaling pathway (Yu et al., 2022).

An additional and noteworthy finding was the modulation of hepatic glucose transporters following EMPA treatment. Although EMPA is primarily known as a selective renal SGLT2 inhibitor, our data revealed no significant changes in hepatic SGLT2 or SGLT1 mRNA and protein expression between EMPA-treated and untreated Zucker rats. Interestingly, this lack of change was accompanied by a significant upregulation of GLUT2 protein levels in the liver of EMPA-treated animals. Our observations imply a potential regulatory effect of EMPA on insulin-mediated glucose transport in hepatocytes, consistent with its insulin-sensitizing and hypoglycemic effects. Interestingly, the same upregulation of GLUT2 by EMPA was found in pancreatic β-cells (Guler et al., 2023). While the underlying mechanisms of this selective glucose transporter modulation are yet to be fully understood, our findings suggest that EMPA modulates hepatic glucose metabolism through pathways that go beyond its established glycosuric action, highlighting its potential as a multifaceted regulator of glucose homeostasis.

Here, beyond glucose metabolism, EMPA positively modulates hepatic lipid homeostasis and markers of mitochondrial function. Indeed, EMPA treatment was associated with reduced expression of CD36 and FoxO1, two key regulators of hepatic steatosis and lipogenesis (Hu et al., 2024). Concurrently, it enhanced the expression of FABP1, PPAR-γ, and its coactivator PGC-1α, indicating a shift toward enhanced fatty acid oxidation and improved mitochondrial energetic adaptation. This metabolic reprogramming was further supported by the upregulation of UCP2 and ABCG1, which play critical roles in mitochondrial function and lipid metabolism (Dikalov et al., 2024). Other experimental and clinical studies have similarly reported that SGLT2 inhibitors reduced hepatic steatosis and improved energy homeostasis by reducing lipid accumulation and enhancing mitochondrial efficiency in metabolic disorders (Jojima et al., 2016; Szekeres et al., 2021; Wei et al., 2021; Androutsakos et al., 2022; Luna-Marco et al., 2024). Notably, the unchanged SREBP1 transcription in liver was consistent with the unmodified cholesterol level following EMPA treatment in this animal model, as previously shown (Aragon-Herrera et al., 2019). Furthermore, our previous data demonstrated that EMPA modified the hepatic metabolome of ZDF rats towards a protective profile by modulating other types of lipids (i.e., increased monounsaturated and polyunsaturated glycerides, phosphatidylcholines, phosphatidylethanolamines, lysophosphatidylinositols and lysophosphatidylcholines) (Aragon-Herrera et al., 2022). Consistently, other authors described that EMPA affects many upregulated and downregulated genes, closely related to hepatic glucose and lipid metabolism, in rodent models of T2DM and NAFLD, by performing RNA-sequencing in liver (Lv et al., 2020; Ma et al., 2021).

Recent studies further support the hepatoprotective effect of EMPA protecting against bile duct ligation-induced liver injury in rats through its antioxidant and anti-inflammatory properties (Shakerinasab et al., 2022). Additionally, EMPA promotes autophagy, reduces endoplasmic reticulum stress, and inhibits hepatocyte apoptosis, slowing the progression of NAFLD in mice (Nasiri-Ansari et al., 2021).

In the liver of EMPA-treated rats, we observed a significant reduction in pro-inflammatory cytokines TNF-α and IL-1β, accompanied by increased expression of M2 macrophage markers such as IL-34, TIMD4, and CD163. These findings highlight EMPA’s capability to modulate hepatic immune responses by promoting a shift from pro-inflammatory M1 to anti-inflammatory M2 macrophages.

Similar effects have been observed in other studies investigating the immunomodulatory properties of EMPA, where it has been shown to modulate macrophage polarization and reduce inflammation in other tissues (Xu et al., 2017; Lu et al., 2022; Xie et al., 2022). Indeed, IL-34 has been implicated in obesity, diabetes and their related disorders including inflammation and IR, contributing to macrophage differentiation and inflammatory response regulation in metabolic tissues, including the liver (Al-Shaebi et al., 2020), and potentially facilitating tissue repair (Chang et al., 2014; Lelios et al., 2020). Moreover, TIMD4 has been associated with enhanced efferocytosis and anti-inflammatory macrophage polarization, which are crucial for resolving obesity-associated chronic inflammation and MASH (Guha Ray et al., 2023; Cao et al., 2024). Meanwhile, CD163, a scavenger receptor primarily expressed on M2 macrophages, has been linked to the resolution of inflammation and metabolic homeostasis (Skytthe et al., 2020). Increased CD163 expression in metabolic tissues has been shown to correlate with improved insulin sensitivity and reduced systemic inflammation in obese animals and patients with MAFLD (Rosso et al., 2019; Schleh et al., 2024). The upregulation of IL-34, TIMD4, and CD163 by EMPA in ZDF rats may influence immune cell recruitment and function, adding another layer to its immunomodulatory properties. Overall, these data indicate that EMPA fosters an anti-inflammatory hepatic microenvironment, which is crucial for resolving chronic inflammation and preventing the progression of MAFLD into fibrosis (Tacke and Zimmermann, 2014; Kazankov et al., 2019).

Consistently, we observed a significant hepatic antifibrotic effect of EMPA, evidenced by reduced levels of TGF-β and type III collagen, confirming previous and recent data from metabolic and/or inflammatory animal models (Abdalla et al., 2023; Elseweidy et al., 2024).

A particularly compelling aspect of our study is the identification of EMPA as a pro-resolving agent in hepatic inflammation. Notably, EMPA treatment led to increased expression of IL-10 and AnxA1, two key mediators of the resolution phase of inflammation and tissue repair (Locatelli et al., 2014; Lurje et al., 2023). These molecules play central roles in dampening chronic inflammation and restoring tissue homeostasis in obesity, diabetes, and other inflammation-driven disorders (Sugimoto et al., 2016; Pietrani et al., 2018; Pitchai et al., 2024). Indeed, IL-10 is a potent anti-inflammatory cytokine known to suppress chronic inflammatory responses and promote tissue repair in metabolic diseases. It improves insulin sensitivity by reducing the production of pro-inflammatory cytokines and limiting macrophage infiltration in adipose tissue, liver, and skeletal muscle (Cintra et al., 2008; Hong et al., 2009; Toita et al., 2016).

Similarly, AnxA1 is a pivotal mediator in the resolution of inflammation, with growing evidence supporting its role in obesity-related IR and MASH (Locatelli et al., 2014; Pietrani et al., 2018). AnxA1 exerts protective effects by inhibiting pro-inflammatory signaling pathways, enhancing macrophage polarization towards the anti-inflammatory M2 phenotype, and mitigating oxidative stress in metabolic tissues (Pietrani et al., 2018).

Our findings reinforce the emerging hypothesis that EMPA, beyond its glucose-lowering effects, actively plays an active role in restoring immune homeostasis within the liver. By modulating macrophage polarization and enhancing specialized pro-resolving mediators, EMPA engages a distinct pro-resolving mechanism that complements its glucose-lowering action. While other oral hypoglycemic agents, such as metformin, exert anti-inflammatory effects primarily via AMPK activation and NF-κB signaling inhibition (Pantazopoulos et al., 2025), EMPA appears to actively promote immune resolution and tissue repair.

## 5 Conclusion

Taken together, our findings provide compelling evidence that EMPA exerts hepatoprotective effects in the context of metabolic syndrome by improving insulin sensitivity, restoring lipid metabolism, enhancing mitochondrial function, and promoting the resolution of inflammation. Through these coordinated actions, EMPA disrupts the vicious cycle triggered by pro-inflammatory macrophage activation, tissue IR and hyperinsulinemia, which are interconnected processes that sustain and amplify metabolic and hepatic dysfunction.

Based on its distinct immunometabolic profile, EMPA should be recognized as a pro-resolving agent in hepatic inflammation, adding a novel dimension to its pharmacological action and positioning it as a promising therapeutic candidate for inflammatory liver diseases associated with metabolic dysfunction. Future studies are needed to elucidate the translational relevance of these findings in clinical MAFLD settings. Furthermore, redefining MAFLD as a systemic metabolic disorder rather than a liver-centric condition, and focusing on novel multifunctional metabolic therapies, such as EMPA, may result more effective in halting MAFLD progression and associated complications.

## Data Availability

The raw data supporting the conclusions of this article will be made available by the authors, without undue reservation.
