# Supplementary figures and images for: Novel insights into the hepatoprotective mechanisms of SGLT2 inhibitor empagliflozin in Zucker diabetic fatty rats
Source: Front Pharmacol. 2025 Sep 23;16:1649630. doi: 10.3389/fphar.2025.1649630 (PMC12500427; doi:10.3389/fphar.2025.1649630)

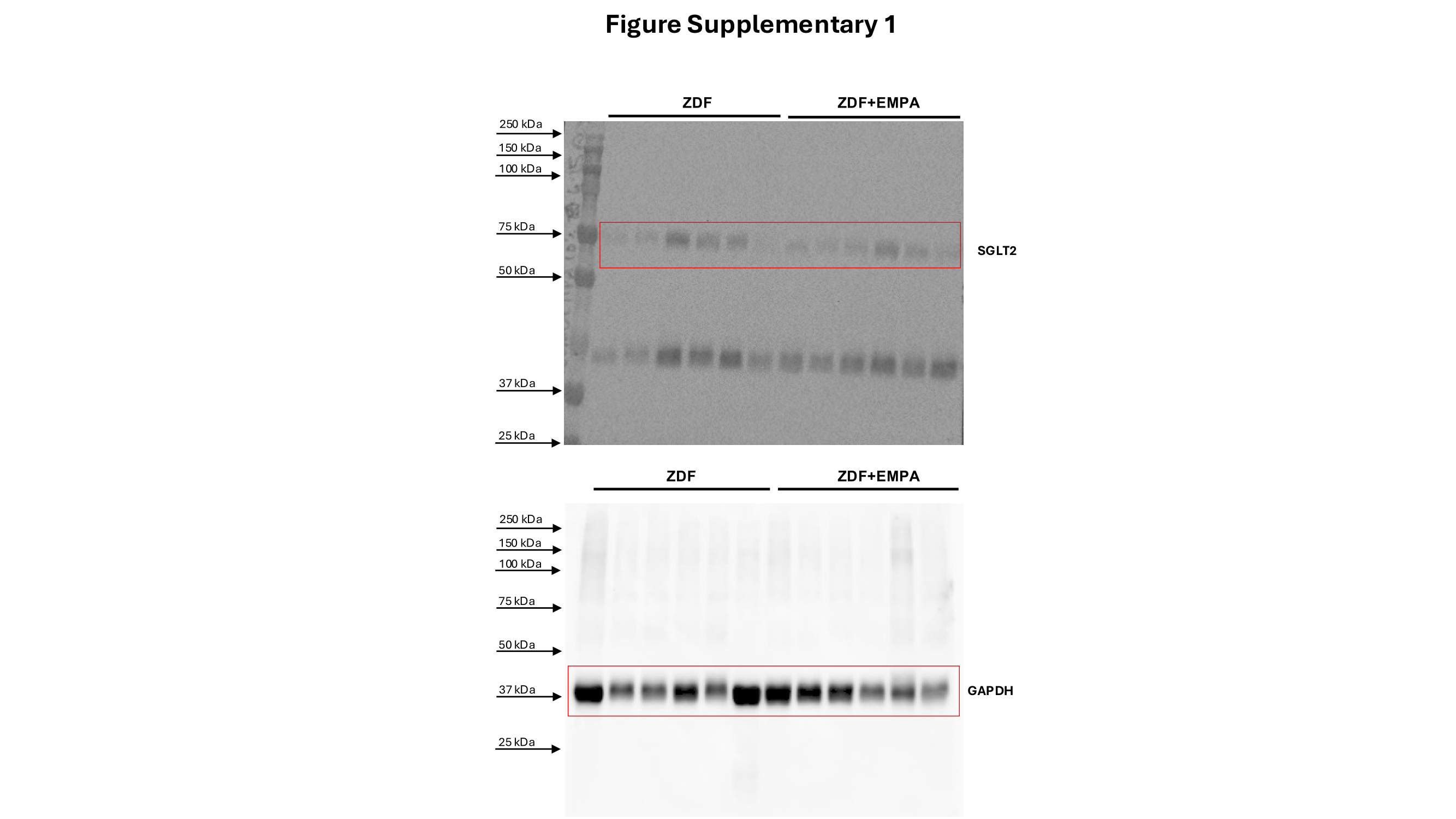

Supplement: Supplementary file 1 [file Image1.tiff]
